# The transformative potential of Social and Solidarity Economy in elder care: social needs, social relations and provisioning systems

**DOI:** 10.3389/fsoc.2026.1805562

**Published:** 2026-06-05

**Authors:** Sofia Adam, Ifigeneia Douvitsa, Maro Pantazidou, Zacharo Vezyri

**Affiliations:** 1 Department of Social Policy, Democritus University of Thrace, Komotini, Greece; 2 Faculty of Sobey School of Business, International Centre for Co-Operative Management, Saint Mary's University, Halifax, NS, Canada; 3 Department of Politics, Centre for Applied Human Rights, University of York, York, United Kingdom

**Keywords:** aging, care, elder care, Social (and) Solidarity Economy, social reproduction theory, social needs, global care chains

## Abstract

Across advanced capitalist economies, elder care has emerged as a critical fault line of social reproduction, as population aging, welfare retrenchment, and care commodification intensify pressures on households and care systems, while dominant responses increasingly rely on market-based provision and global care chains that redistribute social reproductive burdens across borders. Although Social and Solidarity Economy (SSE) initiatives are often advanced as alternatives, their role in elder care remains theoretically underdeveloped and is frequently framed either as compensatory to welfare withdrawal or as inherently transformative. This paper explores the transformative potential of SSE practices in elder care without presuming their emancipatory character. It develops an exploratory conceptual framework drawing on Social Reproduction Theory (SRT) and care-centered approaches. Rather than integrating them into a unified framework, the paper mobilizes SRT and care approaches as distinct lenses that illuminate both the political-economic conditions under which care is devalued and the relational, affective, and ethical labor through which it is enacted and negotiated. The analysis reconceptualizes elder care as a contested system of social needs and social relations shaped by power asymmetries and institutional configurations, shifting attention from organizational forms to the capacity of SSE initiatives to repoliticize social needs and reconfigure social relations. Adopting an ecosystemic perspective that integrates care recipients, caregivers, and families or communities, the paper argues that SSE initiatives contribute to more collective and democratic forms of social reproduction only under specific institutional and relational conditions, while remaining constrained by broader political-economic structures.

## 1 Introduction

Across advanced capitalist economies, elder care has emerged as a critical fault line of contemporary social organization. Population aging, the erosion of public welfare infrastructures, and the increasing commodification of care have intensified pressures on households and care systems alike (Dowling, 2021; Fraser, 2023). These pressures are often addressed through individualized and market-mediated arrangements, including the expansion of global care chains, which rely disproportionately on migrant labor and reproduce inequalities across class, gender, and migration status (Benería, 2008; Hochschild, 2014). Elder care thus constitutes a key site in which the contradictions of contemporary social reproduction become visible.

This paper intervenes in debates at the intersection of Social Reproduction Theory (SRT), care-centered approaches, and Social and Solidarity Economy (SSE) scholarship. Within SRT, a substantial body of work has analyzed how capitalist economies systematically devalue and externalize life-sustaining activities while structurally depending on them (Bhattacharya, 2017a; Fraser, 2023). In parallel, care-centered approaches have foregrounded relationality, interdependence, and the ethical and political dimensions of sustaining life (Puig de la Bellacasa, 2012; Mol, 2008; Tronto, 2013). At the same time, this literature encompasses diverse strands with different analytical emphases, ranging from care ethics to care regimes and infrastructural perspectives. Despite increasing cross-referencing, the relationship between these bodies of work remains marked by analytical tensions and distinct epistemological starting points (Guérin et al., 2021a; Farris, 2025).

At the same time, SSE initiatives in care—such as cooperatives, community-based organizations, and collective arrangements—have been increasingly discussed as potential alternatives to both marketized and state-centered models of provision (Laville, 2010; Utting, 2015). However, existing scholarship tends to approach SSE either instrumentally, as a compensatory response to welfare retrenchment, or normatively, as an inherently transformative alternative (Adam, 2025; Marques, 2014). This dual framing risks obscuring the conditions under which SSE initiatives may reproduce, rather than transform, existing inequalities and power relations.

Against this backdrop, this paper develops a conceptual framework for analyzing the transformative potential of SSE practices in elder care without presuming their emancipatory character. Rather than synthesizing SRT and care approaches into a unified framework, it mobilizes them as distinct analytical lenses that illuminate different dimensions of the same empirical field: the structural organization of social reproduction on the one hand, and the relational and affective dimensions of care on the other.

The central contribution of the paper lies in reconceptualizing elder care as a contested system of social needs and social relations. It proposes that the transformative potential of SSE initiatives should be assessed along two interrelated dimensions: first, their capacity to repoliticize social needs by making visible unmet, latent, and relational needs that are marginalized under dominant institutional arrangements; and second, their capacity to reconfigure social relations within care provisioning systems, particularly relations between care recipients, caregivers, and families or communities.

To capture these dynamics, the paper introduces an ecosystemic perspective that situates care within a multi-level configuration of interdependent actors and institutions. This perspective enables the analysis of how needs and power relations are negotiated across interconnected levels, from everyday interactions to welfare regimes and transnational care arrangements.

By situating SSE initiatives within these intersecting dynamics, the framework contributes to ongoing debates in three ways. First, it clarifies how SRT and care-centered approaches can be productively combined without collapsing their analytical differences. Second, it offers a way of analyzing SSE not as an external alternative to capitalism, but as a contested field embedded within it. Third, it provides conceptual tools for examining when and how alternative elder care arrangements may contribute to more collective, democratic, and socially just forms of social reproduction.

The paper proceeds as follows. The next section outlines the conceptual evolution of social reproduction and identifies its key analytical commitments. This is followed by a discussion of care-centered approaches and a systematic comparison with SRT. The paper then examines elder care as a central node of the contemporary social reproduction crisis, with particular attention to global care chains. The final section develops the proposed framework and assesses the transformative potential and limitations of SSE initiatives in elder care.

## 2 Conceptual frameworks: social reproduction and/or care

### 2.1 Social reproduction theory: structural conditions of care devaluation

Social Reproduction is a concept marked by analytical openness and internal debate (Bezanson, 2006; Fernandes et al., 2023), famously described as “the fleshy, messy, and indeterminate stuff of everyday life” (Katz, 2001, p. 711). Feminist political economy has developed Social Reproduction Theory (SRT) to explain how capitalist societies systematically organize, devalue, and externalize the activities required to sustain life.

Early feminist interventions linked women's oppression to unpaid domestic labor, most notably in the domestic labor debates (Benston, 1997 [1969]; Dalla Costa, 1972; Federici, 1974; Fortunati, 1995 [1981]). While these debates centered on whether unpaid domestic labor produces capitalist value, later contributions—drawing on critiques by Black feminist scholars (Davis, 1981) and scholars from the Global South—shifted attention away from value production toward the broader social relations through which labor power is reproduced.

Building on Vogel's (2013 [1983]) unitary theory, contemporary SRT converges around two key claims: first, social reproduction cannot be reduced to unpaid domestic labor; second, unpaid reproductive labor does not produce value in the capitalist sense (Bhattacharya, 2017a; Rey-Araújo, 2023). Labor power is a peculiar commodity that is not produced capitalistically, and its reproduction therefore occurs largely outside the direct circuit of capital, even though it remains indispensable to value production (González and Neton, 2014; Bhattacharya, 2017b).

This analytical distinction between production and social reproduction is not a transhistorical separation but one generated by the capital relation itself. Capitalism relegates many life-sustaining activities to a devalued “outside” of value, while structurally depending on them (Weeks, 2011; Ferguson, 2020). Subsequent work has expanded social reproduction beyond labor power reproduction to include the institutions, relations, and ecological conditions that sustain life across generations (Brenner and Laslett, 1991; Bakker and Gill, 2003; Bezanson, 2006).

For elder care, SRT is particularly illuminating. It explains why care for older people—especially because it does not directly contribute to the reproduction of labor power—is among the most devalued forms of social reproduction, and why its organization is repeatedly shifted onto families and/or informal arrangements. At the same time, SRT shows that these arrangements are historically contingent outcomes of welfare restructuring, gendered labor divisions, and financialized capitalism, rather than natural responses to demographic aging (Fraser, 2023). In other words, SRT helps us reframe the elder care crisis as an outcome of capital's structural contradictions rather than as a demographic pressure.

However, while SRT excels at diagnosing structural devaluation, commodification, and exploitation, it is less attentive to the relational, affective, and experiential dimensions through which care is enacted and negotiated in everyday life—dimensions that are central to elder care.

### 2.2 Care approaches: ethics, relationality and collective organization

Care scholarship is not a theoretically unified field but encompasses diverse strands with distinct genealogies, analytical foci, and political orientations. A common denominator is their attention to dimensions of care that tend to be bracketed in structural political economy analyses. For the purposes of this paper, we selectively mobilize three strands: care ethics and the related ontological turn toward interdependence, care regime approaches, and perspectives that conceptualize care as infrastructure and/or as commons.

Care ethics established care not simply as a set of activities directed at dependent populations, but as an orientation grounded in relationality and interdependence. Early foundational contributions (Gilligan, 1982; Noddings, 1984) argued that care constitutes a distinct mode of moral reasoning attentive to relations and context rather than abstract principles. While this work remained vulnerable to feminized or moralized readings of care, subsequent work (Fisher and Tronto, 1990; Tronto, 2013) reframed care as a social and political practice central to democratic life. A related development is the “ontological turn,” which conceptualizes care as a fundamental condition of existence, emphasizing vulnerability and interdependence as constitutive of social life (Kittay, 1999; Butler, 2004; Mol, 2008; Puig de la Bellacasa, 2012; Baraitser, 2017).

Care regime approaches, spanning feminist political economy and welfare state analysis (Dowling, 2021; Folbre, 2006; Rai, 2024), situate care within economic structures and institutional configurations. They highlight the dual character of care as both affectively motivated and shaped by material constraints, and analyze how care is distributed across state, market, family, and community, including through frameworks such as the care diamond (Razavi, 2007). More critical contributions emphasize processes of commodification, corporatization, and familialization (Farris and Marchetti, 2017), as well as the need to connect interpersonal, national, and global dimensions of care (Williams, 2018).

Finally, perspectives on care as infrastructure and as commons foreground its material and collective dimensions. The infrastructural approach conceptualizes care as a foundational condition for the reproduction of social life (Hall, 2020; Binet et al., 2023), while care-as-commons approaches emphasize collective organization and the progressive decommodification of care (Federici, 2019; Dengler and Lang, 2022; Sanchez, 2023).

Across these strands, care scholarship foregrounds the ambivalence of care: while it can be enabling and meaningful, it is also demanding and potentially exploitative, particularly under conditions of inequality (Murphy, 2015; Baraitser, 2017). Care approaches emphasize interdependence while remaining attentive to power asymmetries structured by class, gender, and migration status (Jochimsen, 2003).

For elder care, these perspectives are crucial in making visible needs often marginalized in biomedical and market-centered models, such as companionship, autonomy, and social participation. At the same time, when detached from political economy, care-centered approaches risk moralization or depoliticization, potentially obscuring exploitation and reproducing idealized notions of care (Guérin et al., 2021a; Farris, 2025). Taken together, these strands inform the relational and normative dimensions of the analytical framework developed in this paper.

### 2.3 Convergences and tensions between social reproduction theory and care approaches

For the purposes of this paper, it is essential to clarify both the convergences and the tensions between SRT and care-centered approaches, as these divergences have significant implications for the study of elder care.

A common way of relating the two perspectives is through a genus-species distinction, in which care practices are treated as a subset of social reproduction activities, particularly those involving affective relations. However, this framing is analytically limited. Expanded conceptions of care include practices that do not necessarily involve emotional attachment (e.g., cleaning), while affective engagement is increasingly required across a wide range of service occupations (Hochschild, 1983). Conversely, as (González and Neton 2014) argue, reducing social reproduction to specific activities such as childrearing weakens its analytical power. Rather than positing a hierarchical relationship, it is more analytically productive to conceptualize SRT and care-centered approaches as distinct yet complementary perspectives applied to overlapping empirical terrain. Supplementary Table 1 summarizes the key analytical distinctions between these approaches.

SRT is primarily concerned with explaining how capitalism structurally produces and regulates the separation—and interdependence—between production and reproduction. Treating capitalism as a systemic totality, it foregrounds social reproduction as a key terrain of exploitation, domination, and conflict. Care approaches, by contrast, emphasize relationality, interdependence, and the plurality of practices that sustain life, advancing care as a normative and political principle in its own right.

These different orientations shape their respective emphases. SRT examines how production and reproduction are articulated across regimes of capitalist regulation, identifying social reproduction as a site of resistance, refusal, redistribution, and regulation (Hester and Stronge, 2025; Nadasen, 2023). Care approaches focus more strongly on interpersonal relations and on political projects that position care as a guiding framework for social re-foundation. While SRT exposes the exploitative and power-laden character of social reproduction, care-centered approaches—despite their attention to class, gender, ethnicity, and physical capacity—risk sliding into consensual or even fetishized accounts of care if structural relations of exploitation and domination are insufficiently theorized (Guérin et al., 2021a; Farris, 2025).

At the same time, SRT's emphasis on labor aims to denaturalize the historical assignment of reproductive activities to women, particularly migrant women. Because unpaid reproductive labor is non-productive from the standpoint of capital, it is systematically devalued; making this devaluation visible is widely regarded as a precondition for redistribution and collective reorganization. Care approaches locate this devaluation not only in labor relations but also in the dominant value systems of capitalist modernity, conceptualizing care as an ethical-political disposition capable of sustaining broader collective projects such as caring communities. This distinction is analytically productive, but politically ambivalent.

A further divergence concerns the institutional organization of care. In SRT, the interrelation of family, state, market, and third sector is structural and historically dynamic; these are not autonomous pillars but interconnected components of capitalist social formations whose configurations define different regimes of social reproduction (Fraser, 2023). Care approaches often rely on the “care diamond,” which distinguishes family, state, community, and market as governed by different logics—householding, redistribution, reciprocity, and exchange. While analytically useful, this framework risks idealizing plural provision if systemic interdependencies are not fully theorized. As feminist political economy emphasizes, neither the state nor the family exists outside capitalist social relations; both are historically specific and shaped by internal antagonisms (Munro, 2019; Doyle Griffiths, 2020).

These analytical differences also inform how the present conjuncture is understood. Fraser (2023) conceptualizes it as a crisis of social reproduction, in which market expansion undermines its own extra-economic conditions of existence. Nadasen (2023), however, cautions that crisis narratives may obscure the long-standing reproductive crises experienced by marginalized populations and underestimate capitalism's capacity to profit from care deficits, as evidenced by the expansion of the care economy. Care crisis discourses, moreover, often privilege middle-class anxieties while sidelining the experiences of caregivers, who are disproportionately migrant women.

SRT is indispensable for elucidating the structural mechanisms through which care is systematically devalued, yet treating care as either inherently virtuous or inherently exploitative constrains analytical and political imagination. Research on elder care therefore requires a synthetic framework that keeps commodification and structural devaluation in view while attending to the relational and affective labor through which people sustain one another under precarious conditions.

As Baraitser (2017), 2018) and (Mol (2008) remind us, care is an inherently ambivalent practice, involving endurance, attachment, frustration, and the difficult work of staying in relation. Holding this ambivalence—and organizing it collectively—is a key condition for imagining alternatives to existing care arrangements. Accordingly, this paper approaches care as simultaneously labor and relation, constraint and possibility: structured by class, gender, and migration regimes, yet remaining a site of social transformation.

Taken together, SRT and care approaches provide the analytical foundations for the exploratory framework developed in this paper. SRT clarifies why elder care is systematically devalued and unevenly organized, while care approaches illuminate how needs are experienced, articulated, and negotiated within everyday relations. Building on this dual perspective, the following sections analyze SSE initiatives in elder care not as inherently transformative alternatives, but as contested spaces in which systems of social needs and social relations may be partially reconfigured under specific historical and institutional conditions.

## 3 Social and solidarity economy in social reproduction: a field of contestation and struggle

Building on the preceding discussion, this section situates SSE practices within the contested terrain of social reproduction. From an SRT perspective, SSE initiatives cannot be understood as external to capitalism, nor as inherently emancipatory alternatives. From a care perspective, however, they may constitute sites where relational practices, collective responsibility, and alternative articulations of needs are experimentally reorganized. Holding these lenses together allows SSE to be analyzed as a field of struggle over the organization of care, social needs, and social relations, rather than as a coherent sector or normative project.

SSE combines two related but distinct traditions: the social economy and the solidarity economy (Adam, 2018). Both refer to economic activities that do not follow the dominant capitalist logic of profit maximization and instead emphasize democratic governance, collective ownership, and social objectives (Laville, 2010; Utting, 2015). While the social economy historically focused on organizational forms such as cooperatives, mutual aid societies, and associations, the solidarity economy extends this focus to a broader political project aimed at social transformation. SSE thus encompasses a heterogeneous set of entities, including cooperatives, associations, foundations, social enterprises, and informal self-help initiatives (ILO, 2022).

From the standpoint of SRT, the significance of SSE in care lies not in its presumed autonomy from capitalism, but in its location within the sphere of social reproduction, traditionally associated with state responsibility and familial obligation. The emergence of SSE initiatives in care must therefore be situated within broader processes of welfare retrenchment, commodification, and the privatization of social risks under financialized capitalism. In this sense, SSE practices are shaped by the same structural pressures that generate the care crisis itself, and may function simultaneously as coping mechanisms, survival strategies, and sites of contestation.

Polanyian approaches conceptualize SSE practices as hybrid arrangements articulating multiple principles of economic integration—reciprocity, redistribution, market exchange, and householding (Laville and Eynaud, 2019). These principles do not map neatly onto distinct institutional spheres but coexist within and across organizations. This analytical framework challenges totalizing accounts of capitalist dominance by recognizing the persistence of plural economic logics, even in market-dominated societies. SSE initiatives are thus defended not on explicitly anti-capitalist grounds, but because they enable experimentation in meeting social needs and expanding democratic participation.

A similar orientation informs feminist analyses of SSE in social reproduction. Drawing on empirical cases from the Global South, Guérin et al. (2021a) conceptualize SSE through an epistemology of possibilism, emphasizing its capacity to generate social relations that are neither purely domestic nor capitalist. They identify processes through which SSE may contribute to the reorganization of social reproduction: the communalization of care, the extension of social reproduction to encompass human and non-human life, the material and symbolic revaluation of reproductive activities, and the development of hybrid provisioning arrangements based on equality and democratic governance (Guérin et al., 2021b).

From an SRT-informed perspective, however, such claims require careful qualification. Critics argue that SSE practices are deeply embedded in capitalist social formations and often emerge as responses to neoliberal austerity rather than as challenges to it (González and Neton, 2014; Del Re, 2015). Fraser (2023) cautions that community-based and cooperative initiatives can function as integral components of capitalism's adaptive strategies, absorbing social reproductive costs while leaving structural power relations intact. Market-oriented social enterprises may even facilitate welfare privatization and undermine the universality of social rights (Adam, 2018; Marques, 2014). From this viewpoint, social reproduction struggles must remain oriented toward claims for public funding, universal access, and collective responsibility (Ferguson, 2020).

This paper adopts neither a celebratory nor a dismissive stance toward SSE. Instead, consistent with the combined SRT-care framework, SSE initiatives are analyzed as ambivalent and contested arrangements whose transformative potential depends on how they intervene in two analytically distinct but interrelated dimensions: (a) systems of social needs, and (b) social relations within care provisioning systems. SSE practices matter not because they revalue reproductive labor in capitalist terms—since value itself remains a problematic horizon—but because they may disrupt dominant logics governing what counts as a legitimate social need and who is recognized as a legitimate social subject within care systems.

Regarding systems of social needs, capitalism shapes both the production and satisfaction of needs in structurally exclusionary ways. It leaves many needs unmet due to insufficient purchasing power; renders invisible needs that cannot be articulated through market exchange; privileges modes of need satisfaction that facilitate capital valorization; and systematically suppresses collective systems of provisioning (Adam, 2026). These dynamics are particularly visible in elder care, where needs related to companionship, autonomy, and social participation are marginalized in favor of narrowly defined service outputs.

SSE initiatives can potentially intervene in this terrain by making unmet and latent needs visible, articulating alternative understandings of care beyond biomedical and market frameworks, and experimenting with collective modes of provisioning. Whether such interventions remain localized coping strategies or contribute to broader politicization depends on their institutional embedding and relations with state actors.

With regard to social relations, the organizational form of SSE initiatives plays a crucial role in reshaping power asymmetries among actors involved in care provisioning. Drawing on this insight, the paper examines SSE practices along four analytical axes: purpose, membership composition, scope of activities, and legal form (Supplementary Table 2). These dimensions affect who participates in decision-making, how care labor is recognized and compensated, and how conflicts between care recipients, providers, and members of the family and community are negotiated.

With regard to their purpose, SSE entities can be distinguished between those primarily oriented toward the collective benefit of their members, often through cooperative arrangements or mutual assistance schemes, and those oriented toward broader social benefit, such as foundations, associations, and non-governmental organizations (NGOs). The latter typically focus on rights advocacy and the support of vulnerable groups. A third category combines collective and social benefit. A paradigmatic example is provided by Work Integration Social Enterprises (WISEs), which often operate on a cooperative basis and simultaneously pursue the collective benefit of their members—who typically belong to vulnerable groups—and the wider social benefit of the communities in which they operate (ILO, 2016).

Membership composition constitutes a second critical dimension. Along this axis, it is possible to distinguish between single-stakeholder organizations, composed of a single category of actors (e.g., workers-only or users-only), and multi-stakeholder organizations, which broaden participation to include workers, users, local actors, suppliers, or municipal authorities (iCareCoops, 2017). Multi-stakeholder governance reflects the interdependent character of provisioning systems and institutionalizes this interdependence within organizational structures (Girard, 2022).

Significant variation is also observed with respect to the scope of activities. Some SSE entities focus exclusively on care provision, either by delivering highly specialized services of limited scope or by adopting holistic approaches that encompass a wide range of services, from prevention and psychological support to creative and social activities. Other entities operate primarily in different sectors—such as agricultural or credit cooperatives—while developing complementary care-related activities by leveraging existing organizational infrastructures and membership bases. Early health cooperatives in Japan offer a well-documented example, having historically emerged from agricultural cooperatives seeking to meet the care needs of their members (Girard, 2022).

Finally, SSE entities display considerable diversity in terms of legal form. These range from informal, community-based collectives—often with a radical or experimental orientation, such as time banks created by and for community members, where time functions as the unit of exchange for care and support services (Murtagh, 2017)—to fully institutionalized legal entities. The latter may originate from stakeholder groups, evolve from informal initiatives that gradually acquire legal status, or be established by existing organizations creating autonomous entities to integrate care activities more systematically. International experience highlights a wide array of legal forms, including worker cooperatives, user cooperatives, multi-stakeholder cooperatives, mutual aid societies, associations, foundations, and social enterprises (either as a legal form or legal status). In some cases, more conventional legal forms—such as limited liability companies—are adopted to ensure legal security or reduce administrative burdens (ILO, 2016). Challenges frequently arise in contexts where cooperative law does not explicitly recognize multi-stakeholder cooperatives, requiring careful statutory design around governance, representation, and decision-making in order to ensure equitable treatment across different member categories (iCareCoops, 2017).

Consequently, the combined perspective of SRT and care-centered approaches brings to the fore both exploitative dynamics and relational commitments that permeate SSE initiatives in elder care. These initiatives are structurally constrained by precarious funding, gendered labor markets, migration regimes, yet sustained by mobilizing affective ties, trust, and collective responsibility. Their transformative potential cannot be assessed abstractly, but only in relation to specific welfare regimes, regulatory frameworks, and historical trajectories of social reproduction.

This inherent ambivalence is not a weakness of SSE analysis but its analytical starting point. In this light, SSE emerges as more than just a set of alternative organizational forms that formalize care work and provide care services. Its transformative potential is positioned in its capacity to open new spaces for collective experimentation, where prefigurative care practices emerge within structural constraints and limitations. In this regard, SSE may contribute to re-define social needs, social relations and the elder care itself beyond narrow service outputs and market-compatible logics. This potential is especially relevant in the context of global care chains, discussed in the next section, where elder care deficits are managed by narrowly defining existing care needs and reproducing unequal social relations across (and within) national borders.

## 4 Elder care and global care chains: structural fixes and displaced crises

Elder care constitutes a privileged site in which the contemporary crisis of social reproduction becomes visible. In aging societies of the Global North, increasing life expectancy combined with declining fertility rates has intensified care needs at the very moment when public welfare provision is retrenching and labor markets are becoming more demanding. At the same time, higher female labor-force participation, urbanization, and the erosion of extended family and community networks have narrowed the capacity for informal care, producing widespread care gaps.

From a SRT perspective, these developments do not reflect a demographic inevitability but a structural contradiction of capitalism. As Federici (2012) argues, the elder care crisis emerges not from aging itself, but from the capitalist devaluation of reproductive labor and the treatment of older people as “non-productive” subjects—perceived as consuming value without contributing to its production. Extended life expectancy under capitalism thus frequently comes into tension with quality of life, generating experiences of isolation, vulnerability, and social exclusion.

A feminist political economy approach further highlights how elder care inequalities accumulate across the life course. Gendered labor markets, interrupted employment trajectories, and unequal access to social protection result in older women both providing more care and receiving less of it in later life (Stark, 2005; Kotsadam, 2011). Single older women outnumber men not only due to longevity but also due to patriarchal marital patterns, while their lower lifetime earnings constrain access to paid care. Elder care, therefore, cannot be understood apart from broader regimes of class and gender (Estes et al., 2003).

One dominant response to these contradictions has been the expansion of migrant care work, conceptualized through the framework of global care chains (Benería, 2008; Hochschild, 2014; Yeates, 2012). Global care chains describe transnational arrangements in which care deficits in wealthier countries are addressed through the labor of migrant women from poorer regions, creating a series of interconnected care substitutions across households, communities, and countries.

From an SRT perspective, global care chains function as a spatial fix to the crisis of social reproduction. They temporarily alleviate care shortages in receiving countries while displacing reproductive burdens onto migrant workers and their communities of origin. Care labor is extracted from contexts where social reproduction is already under-resourced, generating what has been described as “care drain” (Bettio et al., 2006) or emotional imperialism (Hochschild, 2014). These dynamics are particularly pronounced in Southern European welfare regimes, where familialism and informal employment intersect with large-scale reliance on migrant care labor (Lyberaki, 2011; Gottschall, 2023).

Care approaches complement this analysis by foregrounding the relational and affective dimensions of these arrangements. Migrant caregivers often provide intimate, emotionally demanding care under conditions of precarity, isolation, and limited labor protections. At the same time, care chain narratives that emphasize the “transfer” or “extraction” of love risk re-naturalizing care as women's work, reinforcing stereotypes about migrant women's moral dispositions, and obscuring the diversity of care workers' identities and motivations (Yeates, 2012; Nadasen, 2023).

Several limitations of the global care chains framework are therefore salient for the present analysis. First, framing the issue as a competition between middle-class (often white) women in the Global North and working-class (often non-white) women from the Global South obscures the structural reasons why care responsibilities initially fell on women and ignores the growing role of migrant men in care services, including men with non-heteronormative sexual orientations (Yeates, 2012). Second, its emphasis on transnational flows risks privileging the nation-state as the primary analytical unit, despite the fact that care chains also operate within national and local contexts, including within the Global South. Third, an exclusive focus on vulnerability may obscure migrant caregivers' agency, strategies of negotiation, and forms of collective organization, even under highly constrained conditions (Lyberaki, 2011; Ambrosini and Hajer, 2023).

Despite these caveats, the global care chains' literature remains essential for understanding how elder care needs are currently met through mechanisms that redistribute care deficits rather than resolve them. From the combined SRT-care perspective adopted in this paper, global care chains individualize responsibility, deepen labor market segmentation, delay public investment in socialized care, and normalize highly unequal relations between care recipients, family members, and migrant workers (Benería, 2008; Yeates, 2012).

In this sense, global care chains exemplify a dominant, but ultimately unsustainable, response to the elder care crisis. They address unmet needs through mainly market-mediated and informal arrangements while leaving invisible relational needs—such as companionship, dignity, and participation—largely unaddressed. At the same time, they exacerbate exploitation and precarity among caregivers, particularly migrant women, whose own needs for social reproduction remain marginalized.

By focusing on elder care, this paper aims to elucidate care inequalities, encompassing older adults, paid caregivers, and family members who coordinate care ecosystems. Including these diverse actors acknowledges competing interests—between family members seeking to minimize care costs and caregivers—and the ambivalence of care itself, situated between love/concern and labor devaluation. Collective negotiation to reorganize these tensions, recognizing the distinct needs and empowerment strategies of each group, represents a central challenge and key area for exploring the transformative potential and limitations of SSE practices.

In this regard, the global care chains' discussion does more than setting up the contextual background. It brings into focus the dominant configuration of elder care under contemporary capitalism—characterized by individualized solutions, market mediation, and unequal social relations—which constitutes the terrain that SSE initiatives must confront and potentially reconfigure.

## 5 Discussion: the transformative potential of SSE practices in elder care

Taken together, Sections 3 and 4 do not simply provide contextual background, but generate the analytical dimensions that structure the proposed framework, namely the redefinition of social needs and the reconfiguration of social relations under conditions of marketization and globalized care provision. Section 3 shed light on the transformative potential of SSE to redefine social needs and reorganize social relations in care provisioning systems, while Section 4 examined global care chains, where individualized, market-oriented solutions and unequal relations emerge as dominant trends. These insights are brought together in Supplementary Table 3, which maps needs and social relations across a care ecosystem. While not limited to SSE initiatives, the framework provides a basis for assessing their transformative potential and limitations. The columns outline key domains of transformation within care needs systems, while the rows specify levels of empowerment across actors. The table is presented as an exploratory analytical tool, intended to support conceptual clarification rather than pre-empt empirical investigation.

The following caveats illustrate the main tenets of this analytical framework.

First, we adopt an ecosystemic approach to elder care that encompasses caregivers, care recipients, family networks, intermediaries, and public authorities. The concept of the ecosystem, originally introduced by Tansley (1935) to denote an ecological system of interdependent elements and their interactions, has been adapted in both care-related and SSE-related literatures. Whereas frameworks such as the care diamond and care regime approaches are useful for mapping the institutional distribution of care across state, market, family, and community, the ecosystemic perspective highlights the dynamic interdependencies, overlapping roles, and negotiated tensions among actors, as well as the ways in which needs and social relations are continuously reshaped across multiple scales. Analytically, the ecosystem concept allows us to move beyond static mappings of care provision (as in the care diamond or care regimes) by capturing the dynamic, relational, and conflictual processes through which needs, responsibilities, and power relations are continuously negotiated across interconnected levels.

In the care literature, the ecosystem perspective conceptualizes social care as an interconnected system of actors, resources, and institutions, moving beyond a focus on isolated components. It enables the integration of multiple levels of analysis—from interpersonal relations to community arrangements, national care regimes, and transnational dynamics—and emphasizes coordination and value co-creation through interactions among actors. At the same time, this literature has been criticized for focusing predominantly on health rather than social care and for insufficiently addressing power relations, inequality, and structural constraints (Burn and Needham, 2023). In the SSE literature, the ecosystem concept refers to the institutional and organizational conditions that enable the emergence and development of SSE practices. It highlights the structural disadvantages faced by SSE initiatives in relation to profit-maximizing enterprises, the institutional bias or indifference toward bottom-up initiatives, and the effects of neoliberal restructuring of social protection. Within this perspective, key actors include SSE organizations, public authorities, support structures, and users or beneficiaries (Fontan and Lévesque, 2023). In this paper, these two strands are brought together: the care literature informs a relational and multi-level understanding of care, while the SSE literature highlights the institutional conditions required for alternative care arrangements to emerge and endure.

This combined perspective foregrounds the structural antagonisms embedded in contemporary elder care systems. Care recipients seek recognition and adequate provision while often reproducing familialist expectations, particularly in Southern European contexts. Caregivers seek recognition, decent work, and labor protections under conditions of precarity. Family members attempt to minimize costs amid welfare retrenchment and marketization. Public authorities shift responsibilities across governance levels without corresponding resources. At the same time, care itself is inherently ambivalent: it is not only an institutional arrangement but also a relational and affective process emerging from everyday interactions. These interactions often exceed contractual logics, as emotional and relational dimensions challenge conventional employer–employee distinctions (Baldassar et al., 2017). Together, these dynamics give rise to lived care ecosystems in which care is continuously negotiated and reconfigured.

Second, the framework emphasizes the systematic mapping and articulation of the needs of older people, as well as those of the broader care ecosystem. The objective is not limited to documenting existing conditions but extends to identifying unmet, latent, and unarticulated needs, thereby making visible emerging forms of need and alternative modes of their satisfaction. This approach addresses both quantitative dimensions (e.g., affordability and access to services, economic security) and qualitative dimensions (e.g., autonomy, dignity, and social participation), with the aim of informing the development of new care practices and policy interventions. In this sense, we deliberately move beyond the biomedical model by incorporating the social and relational dimensions of need and advancing a more holistic understanding of care.

Third, the framework highlights the agency of all involved actors beyond their vulnerability. Portraying migrant women caregivers exclusively as victims is limiting, as such representations foreground vulnerability while obscuring their capacity to act as political subjects. Despite adverse conditions and institutional constraints, migrant caregivers develop everyday survival strategies, collective ties, and micro-practices of adaptation that operate both within and against dominant regulatory frameworks (Ambrosini and Hajer, 2023; Lee et al., 2025). At the same time, care recipients are typically treated as passive beneficiaries, while services focus almost exclusively on the satisfaction of basic daily needs—such as cleaning, personal hygiene, cooking, and feeding. Although this approach addresses immediate concerns, it contributes little to the long-term wellbeing of older people. The absence of activities that foster physical exercise, initiative, and cognitive engagement may accelerate functional decline, exacerbate mental health problems, and restrict opportunities for social participation. In this sense, conventional elder care models fail to promote agency, understood as the capacity for intentional action, which is closely linked to psychological, social, and functional wellbeing. Loss of control is frequently associated with feelings of helplessness and, over time, with isolation and social exclusion (Nummijoki and Engeström, 2010).

Fourth, the variability of organizational forms in SSE practices affects the design and delivery of elder care services. Degrees of user involvement range from passive consumption to co-design and shared governance within SSE entities. In some cases, older people assume dual or multiple roles, acting simultaneously as care recipients and providers, or even as founders of SSE initiatives, mobilizing their skills, experience, and social networks (Murtagh, 2017). Particularly significant is the contribution of retired professionals from health and social care sectors in organizing local care initiatives in rural areas, where access to basic services is limited. By founding or participating in care cooperatives, older people actively contest stereotypes of dependency and instead emerge as key actors in collective initiatives—as volunteers, workers, recipients, and providers. Some initiatives also rely on digital platforms that facilitate personalized matching between providers and recipients, offering services ranging from household support to social companionship and leisure activities. The relationships of trust developed between caregivers and recipients distinguish these initiatives from large-scale institutional providers, which often deliver more impersonal and lower-quality services.

Fifth, empowerment must extend to all actors. An exclusive focus on user needs risks intensifying the exploitation of caregivers. Neoliberal approaches to care governance have historically been accompanied by client-centered models that prioritize the needs of service users, often at the expense of care workers (Nadasen, 2023). The systematic neglect of providers' needs—particularly in contexts where care work is increasingly performed by migrant women under precarious conditions—produces exhaustion and deepens exploitation, often through the internalization of norms of love, altruism, and self-sacrifice. This dynamic risks undermining the care workers' own needs for social reproduction and care, as well as those of their families. On the one hand, caregivers are expected to continuously adapt services to recipients' circumstances, engage in reflexive practice, and experiment with new arrangements responsive to evolving needs (Nummijoki and Engeström, 2010). On the other hand, organizational forms must also empower providers through institutional recognition, secure access to social rights, and the protection of their conditions of social reproduction. Finally, dominant models of elder care frequently ignore the broader familial and social networks surrounding care recipients—networks that could function as coordinators or sources of support for all parties involved. In doing so, they reduce what could be a collective and relational arrangement to a narrow dyadic relationship of dependency, often reproducing problematic power dynamics.

Sixth, the transformative capacity of SSE practices in elder care depends crucially on the role of the state. The articulation and satisfaction of care needs are context-dependent: where public or community provision is established, expectations tend to rise; where such structures are absent, older people and their families rely on individualized coping strategies, often resulting in unmet needs. Even where public or state-funded provision exists, insufficient oversight and public spending cuts frequently lead to deteriorating working conditions for care workers, including reduced time per care recipient (Baines and Daly, 2021; Almeida et al., 2022). This, in turn, fuels dissatisfaction among service users and encourages recourse to informal arrangements, often involving uninsured migrant care workers. Under such conditions, low wages and precarious employment are typically even more pronounced (Gottschall, 2023).

In sum, critiques of the commons and SSE under neoliberal governance are not unfounded. If the self-organization of social reproduction is to move beyond functioning merely as a survival strategy, the key political challenge lies in the systematic exploration and critical assessment of alternative provisioning systems. From this perspective, examining different modes of care provision—public provision, community-based provision with public funding, and community-based provision without state support—raises fundamentally political questions that foreground the political character of care arrangements. Central to this inquiry is the unfinished project of the welfare state, understood as a pursuit of universality that guarantees access, quality, accountability, and continuity of care.

Building on the above, SSE practices hold transformative potential to reconfigure existing social relations within elder care ecosystems insofar as they explicitly recognize (a) all stakeholders involved, and (b) the inherent ambiguity of care as both an affective relationship—grounded in trust, attachment, and emotional labor—and a labor relationship that may embed dynamics of exploitation. From this perspective, SSE initiatives should not be idealized as inherently win-win arrangements. Rather, they make visible both explicit and implicit conflicts, as well as the partial and contested possibilities for their reconfiguration within new collective arrangements.

## 6 Conclusions

This paper set out to rethink the transformative potential of SSE practices in elder care without presuming that alternative organizational forms, care practices, or economic arrangements are inherently emancipatory. By situating elder care at the intersection of SRT, care-centered approaches, and SSE scholarship, the paper developed an exploratory analytical framework that conceptualizes transformation not as a sectoral shift or moral achievement, but as a contested reorganization of social needs, social relations, and provisioning systems within historically specific configurations of capitalism. As shown in the preceding analysis of SSE practices (Section 3) and global care chains (Section 4), these dynamics are not abstract but emerge from concrete configurations of marketization, welfare restructuring, and transnational care arrangements.

The analysis demonstrated that elder care constitutes a paradigmatic site of the contemporary crisis of social reproduction. Far from being driven by demographic inevitability, the elder care crisis reflects the structural devaluation of reproductive labor, the retreat and restructuring of welfare states, and the expansion of market-mediated and informal solutions—most notably global care chains. While these arrangements temporarily alleviate care deficits, they displace social reproductive burdens across households, communities, and borders, normalizing unequal power relations between care recipients, family members, and disproportionately migrant care workers. From the combined SRT-care perspective advanced here, such responses individualize responsibility and reproduce existing hierarchies of class, gender, and migration status.

Against this backdrop, the paper's central contribution lies in offering a conceptual framework for assessing SSE practices in elder care that foregrounds ambivalence, contestation, and conditionality. Rather than evaluating SSE initiatives according to normative aspirations, the framework proposes that their transformative potential be examined along two analytically distinct but interrelated dimensions: (a) their capacity to repoliticize social needs—by rendering visible unmet, latent, and relational needs that are marginalized under the dominance of capitalist value and the biomedical model—and (b) their capacity to reconfigure social relations within care ecosystems, including relations between care recipients, caregivers, and family or community members.

By adopting an ecosystemic approach, the framework deliberately moves beyond dyadic models of care and brings into view the structural antagonisms that shape elder care provisioning: between cost containment and care quality, between affective commitment and labor exploitation, and between individualized coping strategies and collective responsibility. In this respect, the ecosystemic perspective advances existing frameworks such as the care diamond or care regime approaches by foregrounding the dynamic, relational, and conflictual processes through which needs, responsibilities, and power relations are continuously negotiated across interconnected levels. Rather than mapping institutional spheres, it captures how care is co-produced and contested within evolving configurations of actors. The notion of a care ecosystem is therefore not merely descriptive but analytically necessary, as it allows us to grasp interdependencies and tensions that remain obscured in frameworks that treat care provision as a set of discrete institutional domains.

Importantly, the framework resists both romanticized accounts of care and reductive narratives of exploitation. Care is approached as simultaneously relational and labor-intensive, enabling and constraining, meaningful and exhausting. Holding this ambivalence analytically is not a weakness but a necessary condition for understanding how care can become a site of collective negotiation and political intervention.

The discussion of SSE practices illustrated how alternative organizational forms may open—under specific institutional and historical conditions—spaces for experimenting with alternative articulations of social needs, social relations, and provisioning systems. Multi-stakeholder cooperatives, community-based initiatives, and hybrid arrangements can potentially redistribute voice, challenge passive recipient models, and recognize caregivers as political subjects rather than invisible labor inputs. At the same time, the analysis underscored that SSE initiatives remain deeply embedded within capitalist social formations. Without stable public funding, regulatory support, and mechanisms of accountability, they risk functioning primarily as survival strategies that absorb social reproductive costs while leaving structural power relations intact.

These findings carry several broader implications. Analytically, the paper contributes to ongoing debates by clarifying how SRT and care-centered approaches can be held together without collapsing one into the other: SRT elucidates the structural conditions under which elder care is devalued and unevenly organized, while care-centered approaches illuminate how needs are experienced, articulated, and negotiated in everyday life. Together, they provide a lens for analyzing SSE not as an external alternative to capitalism, but as a site of struggle and potential reconfiguration.

Politically, the framework cautions against shifting responsibility for elder care onto communities, families, or SSE actors in the absence of robust public commitment. Transforming elder care requires not only organizational innovation but also renewed engagement with the unfinished project of the welfare state—understood as the pursuit of universality, quality, continuity, and democratic accountability in care provision. SSE initiatives can contribute to this project only insofar as they remain connected to broader struggles over social rights, labor protections, and public responsibility for social reproduction.

Finally, the framework developed here is intentionally exploratory. It does not offer a blueprint for reform, nor does it assess SSE organizational forms according to their efficiency. Instead, it provides an analytical tool for empirical research capable of tracing how elder care needs and social relations are negotiated, contested, and partially reorganized across different welfare regimes and institutional contexts. Future research could apply this framework comparatively, examine its relevance beyond elder care to other domains of social reproduction, or explore how SSE initiatives interact with state actors, labor movements, and migrant organizing.

By reframing elder care as a collective, relational, and politically contested field of social reproduction, this paper aims to contribute to more grounded, critical, and analytically precise debates about how care might be reorganized in ways that are socially just, democratically governed, and attentive to both structural constraints and relational possibilities.
