# Supplementary material for: The transformative potential of Social and Solidarity Economy in elder care: social needs, social relations and provisioning systems
Source: Front Sociol. 2026 Jun 5;11:1805562. doi: 10.3389/fsoc.2026.1805562 (PMC13278922; doi:10.3389/fsoc.2026.1805562)
Supplement: Supplementary file 1 [file Table_1.docx]

***Supplementary Material***

# Supplementary Tables

Table 1. Social Reproduction Theory and Care Approaches: Analytical Convergences and Tensions

| **Analytical Dimension** | **Social Reproduction Theory (SRT)** | **Care Approaches** |
| --- | --- | --- |
| **Core analytical focus** | Explains how capitalism produces and organizes the separation—and interdependence—between production and social reproduction within a systemic totality. | Conceptualizes care as a mode of being grounded in an ontology of interdependence and as a normative compass for social transformation. |
| **Primary emphasis** | The articulation of production and reproduction and their regulation across different regimes of capitalist accumulation. | Interpersonal relations, affect, and everyday care practices sustaining social life. |
| **Relation to exploitation and oppression** | Social reproduction is not a sphere of autonomy or “pure affect,” but one structured by relations of exploitation and domination along class, gender, race, ethnicity, and physical capacity lines. | Emphasis on affective dimensions may understate exploitation and power asymmetries, although intersectional care analyses explicitly address these relations. |
| **Conceptualization of labor** | Frames reproductive activities as labor in order to denaturalize their feminization and expose their structural devaluation. Unpaid reproductive labor is non-productive from the standpoint of capital and therefore systematically devalued. | Highlights affect, attachment, and moral responsibility. Care is marginalized not only as labor but also due to dominant capitalist value systems, calling for a broader ethical revaluation of social priorities. |
| **Provisioning systems** | Family, state, market, and third sector (non-profits, associations, etc.) are analytically inseparable and historically reconfigured within capitalist social formations. | Care is organized across four distinct pillars—family, state, community, and market—each governed by different logics (householding, redistribution, reciprocity, exchange). |
| **Understanding of crisis** | Contemporary capitalism is marked by a crisis of social reproduction, as market expansion undermines its own extra-economic conditions of existence, while simultaneously generating new profit opportunities. | Diagnoses a “care crisis,” in which growing segments of the population are unable to care for themselves and others, destabilizing social cohesion. |

Table 2. Typology of SSE organizational forms in social reproduction

| **Analytical Axis** | **Categories** | **Main features** | **Examples** |
| --- | --- | --- | --- |
| **Purpose** | Collective benefit | Benefit to members | Worker co-ops, mutual aid societies |
|  | Social benefit | Benefit stakeholders beyond membership base | Foundations, NGOs, associations |
|  | Mixed purpose | Benefit to members and the wider community | Social co-ops  Work Integration Social Enterprises |
| **Member composition** | Single-stakeholder | Only workers or users | Worker or User co-ops |
|  | Multi-stakeholder | Workers, users, family members, local association, local authorities | Community co-ops, Multi-stakeholder co-ops |
| **Scope of activities** | Care as the main activity | Ranging from home care to prevention, mental health, creative activities | Care co-ops |
|  | Care support as secondary activity | Building on existing membership and infrastructure | Agricultural co-ops, credit co-ops, mutual aid societies |
| **Legal form** | Informal entities | Voluntary work, solidarity, reciprocity, locality | Community care networks |
|  | Institutionalized | Legal accreditation, formal employment relations | Social enterprises, social co-ops, limited liability companies |

Table 3. Transformative Potential of SSE in the elder care: Needs and Social Relations

| **Empowerment \ Needs** | **Unmet Needs** | **Invisible Needs** | **Modes of Need Satisfaction** | **Provisioning System** |
| --- | --- | --- | --- | --- |
| **Care recipients** | Accompaniment, autonomy, access to information | Companionship, activation, recognition of lived experience | Companionship; participation in social and cultural activities; cultural and creative engagement | Municipal funding; social insurance funds; or mixed funding arrangements |
| **Caregivers** | Employment precarity; residence/work permits; professional recognition; language acquisition | Personal time; socialization; care for family and kinship networks | Wage increases; reduction of working hours; standardized leave entitlements; trade union rights | Worker or multi-stakeholder co-ops; allocation of assets by local authorities; framework agreements; public procurement with social clauses |
| **Coordinators / family and community intermediaries** | Trust, safety, support | Possibility of mobility/absence; focus on emotional relationships | Information provision; access to networks of caregivers; financial support | Multi-stakeholder co-ops; supporters’ associations; local committees |
